# Preparation and Properties of Environmentally Friendly Carboxyl Graphene Oxide/Silicone Coatings

**DOI:** 10.3390/ma18092122

**Published:** 2025-05-05

**Authors:** Zhenhua Chu, Jiahao Lu, Wan Tang, Yuchen Xu, Quantong Jiang, Jingxiang Xu

**Affiliations:** 1 Department of Mechanical Engineering, College of Engineering, Shanghai Ocean University, Shanghai 201306, China; zhchu@shou.edu.cn (Z.C.); lucien06123@163.com (J.L.); wtang@shou.edu.cn (W.T.); 17749450088@163.com (Y.X.); 2 CAS Key Laboratory of Marine Environmental Corrosion and Bio-Fouling, Institute of Oceanology, Chinese Academy of Sciences, Qingdao 266404, China

**Keywords:** silicone resin, anti-corrosion, carboxyl graphene oxide, antifouling coating

## Abstract

To address the protective demands of marine engineering equipment in complex corrosive environments, this study proposes an environmentally friendly composite coating based on carboxylated graphene oxide (CGO)-modified water-based epoxy organosilicon resin. By incorporating varying mass fractions (0.05–0.25%) of CGO into the resin matrix via mechanical blending, the microstructure, corrosion resistance, and long-term corrosion kinetics of the coatings were systematically investigated. The results demonstrate that the coating with 0.15 wt.% CGO (designated as KCG15) exhibited optimal comprehensive performance: its corrosion current density (*I*_corr_ = 4.37 × 10^−8^ A/cm^2^) was two orders of magnitude lower than that of the pure resin coating, while its low-frequency impedance modulus (∣*Z*∣_0.1Hz_ = 4.99 × 10^6^ Ω⋅cm^2^) is significantly enhanced, accompanied by improved surface compactness. The coating achieved a 97% inhibition rate against sulfate-reducing bacteria (SRB) through synergistic physical disruption and electrostatic repulsion mechanisms. Long-term corrosion kinetics analysis via 60-day seawater immersion identified three degradation phases—permeation (0–1 day), blockage (1–4 days), and failure (7–60 days)—with structural evolution from microcrack networks to foam-like blistering ultimately reducing by 97.8%. Furthermore, a 180-day atmospheric exposure test confirms the superior weatherability and adhesion of the KCG15 coating, with only minor discoloration observed due to its hydrophobic surface. This work provides theoretical and technical foundations for developing marine anti-corrosion coatings that synergize environmental sustainability with long-term protective performance.

## 1. Introduction

The advancement of marine resource exploitation into deep-sea environments has led to the prolonged exposure of marine engineering equipment to hypersaline and high-humidity conditions, subjecting metallic substrates to the dual threats of severe corrosion and microbially influenced corrosion (MIC) [1]. Studies indicate that global annual economic losses due to marine corrosion exceed hundreds of billions of US dollars [2], while microbial biofilm formation can amplify corrosion rates by over 10-fold. This synergistic interaction between corrosion and biofouling accelerates material degradation, increasing maintenance costs and posing risks of catastrophic failures [3]. Conventional antifouling strategies include mechanical removal, electrochemical methods, ultrasonic techniques, and antifouling coatings [4,5,6]. Among these, organic coatings remain the most prevalent approach by isolating metallic substrates from corrosive environments.

Early marine protection relied heavily on solvent-based coatings, which utilized organic solvents to disperse resin matrices. However, their volatile organic compounds (VOC) pose significant ecological and human health risks [7]. Current mainstream antifouling coatings, primarily tin-free self-polishing systems, employ acrylic resins loaded with zinc or copper additives. These biocides exhibit solubility in seawater, causing ecological harm through bioaccumulation and trophic transfer, ultimately threatening marine biodiversity and human health [8]. In response, eco-friendly antifouling coatings have emerged. Tian et al. [9] developed an RZn-NMA-X coating system by grafting N-hydroxymethylacrylamide (NMA) onto a zinc acrylate copolymer backbone, endowing it with self-crosslinking properties. During hydrolysis, the formation of COO⁻–Na⁺ ion pairs induces polymer chain swelling and surface exfoliation, exposing a refreshed smooth interface that inhibits fouling adhesion. Concurrently, Zn^2+^ release disrupts biofilm ion homeostasis by competing with Ca^2+^ binding sites on microbial membranes. Despite its efficacy, Zn^2+^’s environmental persistence remains controversial. Ding et al. [10] investigated the synergistic antifouling performance of cuprous oxide and graphene oxide (GO) in epoxy coatings. Their results showed that algal adhesion on GO/Cu-modified epoxy coatings was reduced to 13% of unmodified epoxy, with sustained Cu^2+^ release exceeding 100 days. However, Cu^2+^ toxicity risks to marine ecosystems persist.

To address these challenges, researchers are innovating materials and technologies for eco-compatible antifouling solutions. Waterborne coatings, utilizing water as the dispersion medium, eliminate VOC emissions but often fail to meet the durability requirements of deep-sea environments. Waterborne epoxy–silicone resins, combining the hydrophobicity of silicones with the adhesion of epoxies, show promise but suffer from poor interfacial adhesion due to nonpolar group enrichment and inadequate barrier properties [11]. Recent advances leverage nanofillers for performance enhancement. Liu et al. [12] developed an organic–inorganic hybrid system by grafting octadecanoic acid onto epoxy resin with 30 wt.% nano-SiO_2_, achieving a water contact angle > 145° post-abrasion, though nanoparticle rigidity induces stress concentration. Yu et al. [13] enhanced the coating’s mechanical and protective properties by growing TiO_2_ on boron nitride, reporting a 91% increase in tensile strength and three-orders-of-magnitude improvement in the low-frequency impedance modulus compared to pure epoxy.

Graphene oxide (GO), with its two-dimensional lamellar structure and surface functional groups (e.g., hydroxyl, carbonyl), provides physical barrier effects against corrosive agents but faces challenges in resin matrix dispersion [14]. Chen et al. [15] improved barrier performance by incorporating graphitic carbon nitride into epoxy via ultrasonication. Xu et al. [16] engineered a honeycomb-structured surface by evaporating water/ethanol mixtures with GO-modified prepolymers, reducing algal adhesion by 28-fold compared to conventional polyurethane, albeit with complex fabrication. Carboxyl-functionalized GO (CGO) leverages edge carboxyl groups for covalent bonding with polymer matrices while enhancing coating densification [17]. Tian et al. [18] incorporated imidazole-modified CGO (FCGO) into disulfide-containing polyurethane, achieving a 40% tensile stress increase and >80% antibacterial rate. However, quantitative analysis of CGO’s barrier mechanisms and long-term corrosion resistance in waterborne silicone coatings remains limited.

This study addresses this gap by developing an eco-friendly, durable composite coating through the mechanical blending of CGO into waterborne epoxy-modified silicone resin. Systematic investigations evaluate CGO content effects on microstructure, electrochemical behavior, and long-term corrosion kinetics, elucidating its anti-corrosion–antifouling synergy. Atmospheric exposure tests validate practical applicability, providing theoretical and technical foundations for green multifunctional coatings in deep-sea environments.

## 2. Materials and Methods

### 2.1. Test Material

**Figure 1:**
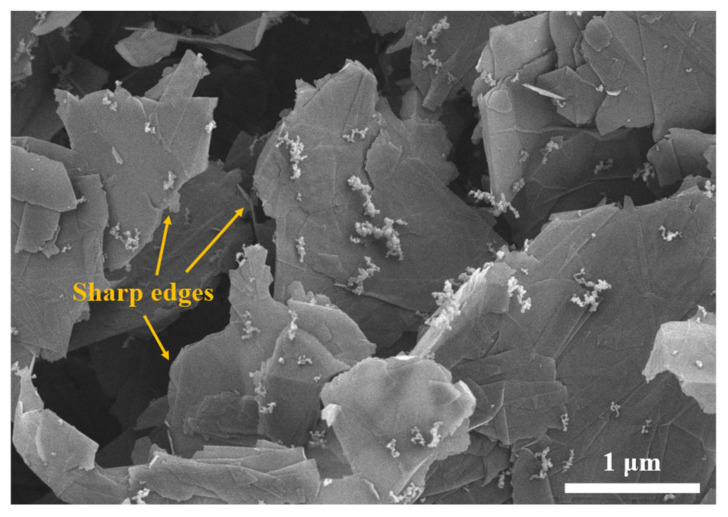
SEM morphology of carboxylated graphene oxide sheets.

The water-based epoxy-modified organosilicon resin emulsion, designated as SH9607, was sourced from Hubei Longsheng Sihai New Materials Co., Ltd. (Zaoyang City, China). Key parameters include a viscosity range of 15–40 mPa·s, a solid content of 50 ± 1%, and an epoxy value of 0.03. The curing agent, γ-aminopropyltriethoxysilane (KH550), with a molecular weight of 221.4 g/mol and purity exceeding 99.1%, was procured from Dongguan Kangjin New Materials Technology Co., Ltd. (Dongguan, China). Carboxylated graphene oxide (CGO) was obtained from Dazhan Nano Co., Ltd. (Shanghai, China), with a lamellar diameter of 9–45 μm and purity of 97%. As shown in Figure 1, the CGO nanosheets display a sharply defined surface morphology characterized by distinct edges and topological irregularities. Aladdin Co., Ltd. (Shanghai, China) supplied anhydrous ethanol (Analytical Reagent grade, AR). For electrochemical testing, water contact angle measurements, and atmospheric exposure tests, the Q235 steel specimens (10 × 10 × 5 mm³), tinplate sheets (150 × 70 × 0.2 mm³), and low-carbon steel plates (150 × 300 × 2 mm³) were respectively purchased from BGD Dalai Instrument Co., Ltd. (Dongguan, China). Sandpapers of various grits (240, 600, and 1000) were sourced from Changzhou Golden Bull Grinding Co., Ltd., Changzhou, China. The sulfate-reducing bacteria (SRB) were provided by the College of Fisheries and Life Sciences, Shanghai Ocean University (Shanghai, China), and the bacterial cultures were commercially sourced from Qingdao Haibo Biotechnology Co., Ltd. (Qingdao, China).

### 2.2. Coating Preparation

**Figure 2:**
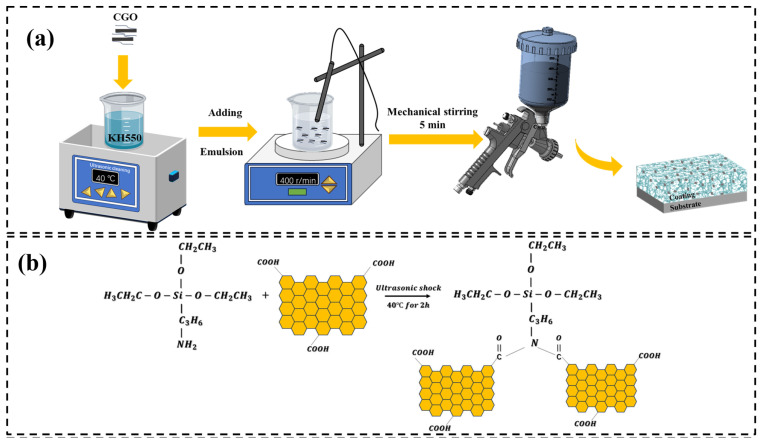
(**a**) Schematic diagram of coating preparation; (**b**) reaction scheme for the synthesis of KGO.

As illustrated in Figure 2a, the schematic diagram outlines the preparation process of the water-based epoxy-modified organosilicon two-component coating. Prior to coating application, the substrate surfaces were mechanically abraded using 240-grit sandpaper to remove oxide layers, followed by sequential polishing with 600-grit and 1000-grit sandpaper. The specimens were then ultrasonically cleaned in anhydrous ethanol and oven-dried for subsequent use. To synthesize the functionalized additive, carboxylated graphene oxide (CGO) was ultrasonically dispersed with γ-aminopropyltriethoxysilane (KH550) at 40 °C for 2 h to produce KH550-functionalized graphene oxide (KGO, Figure 2b). The mixture was then blended with a water-based organosilicon emulsion at a mass ratio of 1:25, mechanically stirred at 400 rpm for 5 min, and aged for 20 min to ensure complete cross-linking between the epoxy-modified organosilicon resin and the KH550 curing agent. Subsequently, the prepared coating was uniformly applied to *Q*235 steel substrates using a spray gun and cured at room temperature for 3 days, resulting in a film thickness of 100 ± 10 μm. The macroscopic morphology and coating properties were then characterized. To determine the optimal loading of carboxylated graphene oxide (CGO), a series of composite coatings were fabricated with varying CGO concentrations (0.05 wt.%, 0.1 wt.%, 0.15 wt.%, 0.20 wt.%, and 0.25 wt.%), designated as KCG5, KCG10, KCG15, KCG20, and KCG25, respectively. The pristine water-based organosilicon resin (WSI) served as the control sample.

### 2.3. Characterization Analysis Method

The surface morphology of specimens was characterized using scanning electron microscopy (SEM, JSM-7500F, JEOL, Tokyo, Japan) operated at an accelerating voltage of 20 kV, with samples subjected to gold sputter coating to enhance conductivity prior to imaging. Fourier-transform infrared spectroscopy (FTIR, Nicolet iS20, Thermo Fisher Scientific, Waltham, MA, USA) was employed to investigate functional group evolution during the powder modification and coating curing processes, with spectral acquisition spanning 400–4000 cm^−1^. For powder samples, the powdered material was mixed with KBr, thoroughly ground, and pressed into transparent pellets. For coatings, the thin-film pressing method was employed. The elements’ valence changes and chemical states were analyzed using X-ray photoelectron spectroscopy (K-Alpha XPS, Thermo Scientific, Waltham, MA, USA). The adhesion strength of the coatings was quantified using an automated pull-off adhesion tester (BGD 500/S, Dalai Instrument Co., Ltd., Dongguan, China) under standardized conditions: a pressurization rate of 0.2 MPa/s, a maximum pressure threshold of 18.00 MPa, and a dwell time of 60 s. Hydrophobic/hydrophilic properties were assessed via water contact angle measurements using a JC2000D3-X contact angle/surface tension analyzer (Shanghai Zhongchen Digital Technology Co., Ltd., Shanghai, China). At the same time, coating thickness was non-destructively determined using a CT400 ultrasonic thickness gauge (Mitutoyo, Kawasaki, Japan). Acridine orange stain was used for fluorescent bacterial staining. The specimens were immersed in 0.1% acridine orange stain for 5 min in a light-free environment, rinsed with PBS buffer, and then placed on slides to be observed under a fluorescence microscope (Leica DMi8, Wetzlar, Germany).

Electrochemical impedance spectroscopy (EIS) measurements were conducted using a Reference 600+ potentiostat/galvanostat (Gamry Instruments, Warminster, PA, USA) configured with a conventional three-electrode system: the coated specimen as the working electrode, a platinum mesh counter electrode, and a saturated calomel electrode (SCE) as the reference. EIS spectra were periodically recorded during immersion testing in artificial seawater (composition detailed in Table 1) across a frequency range of 10^5^–10^−1^ Hz with an AC perturbation amplitude of 10 mV. Data interpretation utilized ZSimpWin 2.0 software for equivalent circuit modeling. Potentiodynamic polarization scans were performed at a sweep rate of 1 mV s^−1^ over a potential window of −0.5 V to +0.5 V vs. open-circuit potential (OCP). Before electrochemical testing, specimens were stabilized in the electrolyte for 1 h to attain steady-state OCP conditions. Corrosion kinetic parameters were derived from polarization curves using potentiodynamic extrapolation methodology.

## 3. Results

### 3.1. Surface Feature

**Figure 3:**
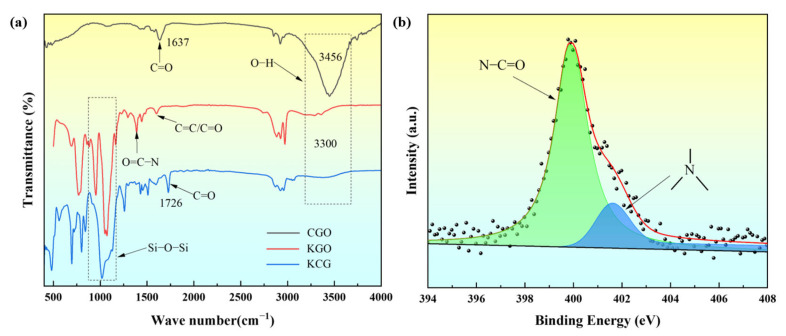
(**a**) FTIR spectra of CGO, KGO, and KCG; (**b**) XPS high-resolution N1s spectrum of KGO.

Figure 3a compares the infrared spectra of carboxyl-functionalized graphene oxide (CGO), silane coupling agent KH550 modified graphene oxide (KGO), and silicone composite coatings (KCG), systematically revealing the evolution of functional groups during the modification process. The characteristic peaks of CGO at 3456 cm^−1^ (O-H stretching vibration) and 1637 cm^−1^ (C=O asymmetric stretching vibration) confirm the presence of carboxyl groups [19]. After modification with KH550, the intensity of the O-H peak in KGO weakens, and the C=O peak disappears, while new peaks appear at 2971/2925 cm^−1^ (C-H vibrations of the KH550 propyl chain), 1600/1342 cm^−1^ (C=O/C-N bonds), and 1164/1071 cm^−1^ (Si-O-C condensation peaks). Notably, the C=O stretching vibration of amide bonds (–O=C-N–) shifts to 1630–1600 cm^−1^ due to conjugation with aromatic C=C systems, partially overlapping with the intrinsic GO C=C vibration at 1600 cm^−1^. In Figure 3b, the deconvoluted N 1s XPS spectrum of KGO reveals two components [20]: –N–C=O (399.8 eV) and ternary amines (401.5 eV), confirming the formation of amide bonds via dehydration condensation between KH550’s amino groups and CGO’s carboxyl groups. Additionally, the Si-O-Si/Si-O-C peak at 1018 cm^−1^ in CGO intensifies, confirming the covalent bonding between KGO and silicone. Furthermore, the emergence of new peaks at 1726 cm^−1^ (C=O) and 1258 cm^−1^ (C-O-C ether bonds) corroborates the cross-linking reaction between KH550 amino groups and epoxy–silicone resin, ultimately forming a stable siloxane–epoxy synergistic network.

**Figure 4:**
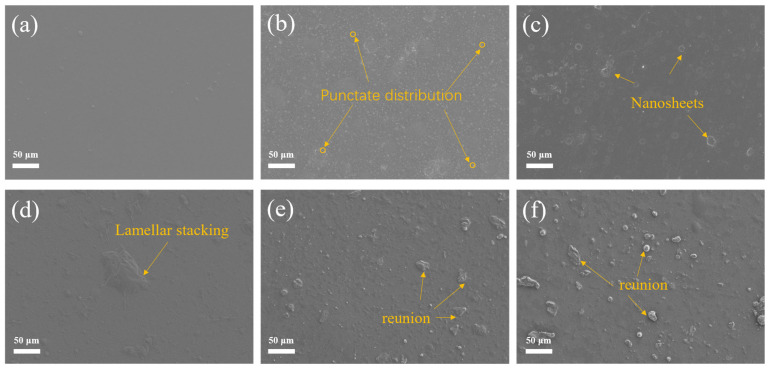
Micromorphology of coatings with different carboxylated graphene oxide content: (**a**) WSI, (**b**) KCG5, (**c**) KCG10, (**d**) KCG15, (**e**) KCG20, (**f**) KCG25.

Figure 4a–f present a comparative analysis of the surface morphology of pure silicone coatings (WSI) and composite coatings with varying CGO contents (0.05–0.25 wt.%) through SEM. The WSI surface appears smooth and featureless (Figure 4a). After the addition of 0.05 wt.% CGO (Figure 4b), uniform nanoscale protrusions are observed, indicating that the KH550 modification allows for the even dispersion of CGO. As the CGO content increases to 0.15 wt.% (Figure 4c,d), the density of protrusions rises, forming a continuous layered structure, which is attributed to the synergistic cross-linking of oriented CGO nanosheets with the siloxane network. However, when the CGO content exceeds 0.15 wt.% (Figure 4e,f), micrometer-scale agglomerates form due to π-π* stacking, resulting in an increase in surface roughness.

### 3.2. Hydrophobicity and Adhesion

**Figure 5:**
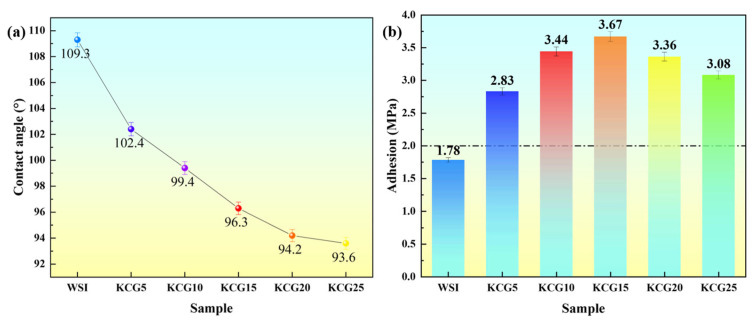
(**a**) Static water contact angle and (**b**) the adhesion of different samples.

Surface wettability is one of the important factors affecting the adhesion of fouling organisms, which directly relates to the antifouling performance of coatings. The static water contact angles of different coatings are illustrated in Figure 5a, revealing significant differences among them: the unmodified silicone coating (WSI) exhibits strong hydrophobic properties (109°), attributed to the low surface energy barrier formed by the directional arrangement of nonpolar methyl groups on its surface [21]; in contrast, the contact angles of carboxyl-functionalized graphene oxide (CGO) composite coatings (KCG series) decrease gradually with increasing amounts of CGO (KCG5: 102°, KCG10: 99°, KCG15: 96°, KCG20: 94°, KCG25: 93°). Although the abundant carboxyl groups (-COOH) on the CGO surface mitigate the hydrophobicity of the silicone resin, all KCG coatings remain in a hydrophobic state (θ > 90°), indicating that they can achieve antifouling properties under dynamic water flow conditions through a synergistic effect of hydrophobicity and shear.

The interfacial adhesion strength of the coating is a key mechanical indicator that ensures its long-term service in complex marine environments. As shown in Figure 5b, the WSI demonstrates insufficient interfacial chemical bonding due to the enrichment of nonpolar groups on its surface [22], resulting in an adhesion strength of only 1.78 MPa. In contrast, the adhesion strength of CGO composite coatings first increases and then decreases with the addition of CGO (KCG5: 2.83 MPa → KCG15: 3.67 MPa → KCG25: 3.08 MPa). This phenomenon relates to the interfacial enhancement mechanism of CGO: when the amount of CGO added is ≤15 wt.%, the carboxyl groups on its surface form a covalent bond network (Si-O-C) with the silanol groups in the silane coupling agent through dehydration condensation, significantly enhancing the interface bonding strength between the coating and the substrate; however, when excess CGO is present (>15 wt.%), the agglomeration effect of the nanosheets leads to stress concentration, which in turn weakens the mechanical properties. It is noteworthy that the adhesion strengths of all KCG coatings exceed the threshold requirement for marine protective coatings (2 MPa), indicating their potential for practical engineering applications.

### 3.3. Antibacterial Activity Evaluation

**Figure 6:**
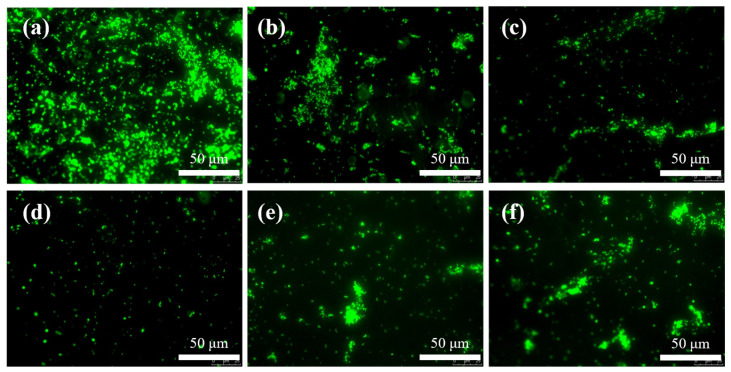
Bacterial adhesion densities on surfaces of distinct samples: (**a**) WSI; (**b**–**f**) KCG5–KCG25, respectively.

Marine microbial corrosion is predominantly (>70%) initiated by sulfate-reducing bacteria (SRB), and inhibiting biofilm formation on coating surfaces can directly impede the secondary adhesion of macrofouling organisms such as barnacles and algae. Figure 6 presents fluorescence microscopy images illustrating the biofouling behavior of distinct coatings after 10-day immersion in SRB suspensions: the pristine silicone coating (WSI) exhibits extensive coverage of viable bacteria (>90%, Figure 6a), indicating insufficient static antimicrobial activity. In contrast, coatings incorporating carboxyl-functionalized graphene oxide (CGO) demonstrate significantly reduced viable bacterial densities, following a non-monotonic trend—KCG15 achieves the lowest bacterial coverage (<3%, Figure 6d), while KCG25 shows partial resurgence (20%, Figure 6f). This phenomenon is attributed to CGO’s dual-action mechanism: at optimal loadings (≤0.15 wt.%), CGO synergistically disrupts bacterial membrane integrity through physical piercing by sharp edges and electrostatic attraction via carboxylate anions. Conversely, excessive CGO introduces heightened surface roughness that promotes bacterial anchoring, leading to an imbalance between bactericidal and anti-adhesion efficacy. Notably, graphene oxide concentrations exceeding 50 μg/mL are required to elicit ecotoxicological effects in aquatic organisms [23]. The CGO release concentration from these coatings in marine environments remains well below this threshold, ensuring compliance with ecological safety standards.

### 3.4. Corrosion Resistance

**Figure 7:**
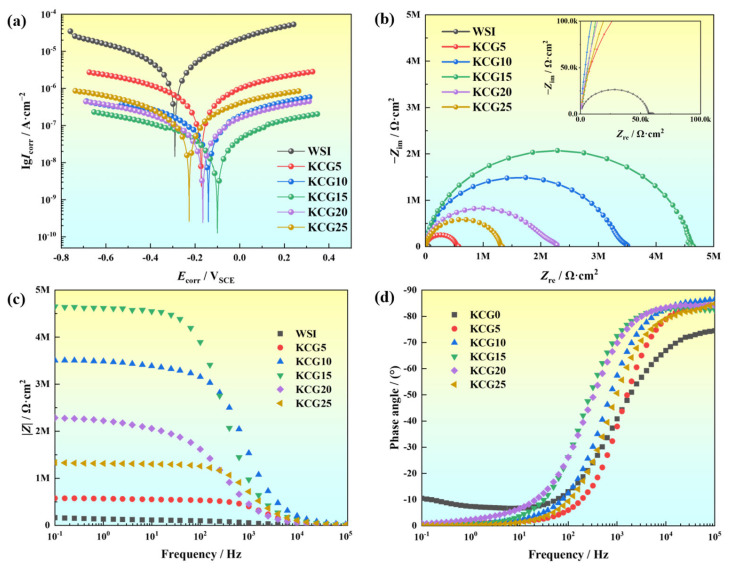
Electrochemical performance of coatings with different carboxylated graphene oxide content: (**a**) polarization curve, (**b**) Nyquist graph, (**c**) Bode modulus |Z| graph, (**d**) Bode modulus frequency angle graph.

The electrochemical performance of the coatings was investigated using polarization curves and electrochemical impedance spectroscopy (EIS). Corrosion behavior was quantitatively analyzed via polarization curves and semi-quantitatively assessed through EIS. Figure 7a presents the Potentiodynamic polarization curves of carboxylated graphene oxide (CGO) coatings with varying CGO content in artificial seawater. The corrosion potential (*E*_corr_) and corrosion current density (*I*_corr_) of the coating systems were calculated using the Butler–Volmer electrochemical kinetics equation to analyze the coatings further [24]. Under identical conditions, higher *E*_corr_ and lower *I*_corr_ values indicate reduced corrosion susceptibility and enhanced corrosion resistance.

Table 2 summarizes the polarization curve fitting parameters for coatings with different CGO contents. The data show that the pure resin (WSI) exhibited *E*_corr_ = −0.291V and *I*_corr_ = 2.05 × 10^−6^ A⋅cm^−2^. All CGO-modified coatings demonstrated *E*_corr_ and *I*_corr_ values superior to those of WSI. Notably, KCG15 achieved the highest *E*_corr_ (−0.101V) and the lowest *I*_corr_ (4.37 × 10^−8^ A⋅cm^−2^). The *I*_corr_ of WSI was 40 times higher than that of KCG15, indicating that KCG15 exhibits the best anti-corrosion performance among all samples.

Figure 7b displays the Nyquist plots of the coatings in artificial seawater. Generally, the radius of the capacitive arc in a Nyquist plot is inversely proportional to the corrosion rate [25]: a larger arc radius indicates higher coating resistance and slower corrosion, whereas a smaller arc radius corresponds to faster corrosion. As shown in Figure 7b, the capacitive arc radii of the coatings follow a distinct trend, KCG15 > KCG10 > KCG20 > KCG25 > KCG5 >> WSI, suggesting that the corrosion resistance of the composite coatings initially improves and then declines with increasing carboxylated graphene oxide (WSI) content. CG15 exhibits the largest capacitive arc radius and the best corrosion resistance. Microscopic morphology analysis reveals that surface roughness increases with higher CGO content, and excessive CGO leads to particle agglomeration on the coating surface. Figure 7c,d present the Bode magnitude and phase plots of the coatings in artificial seawater. The corrosion resistance was evaluated by comparing the low-frequency impedance modulus (∣*Z*∣_0.1 *Hz*_). Compared to CGO-modified composite coatings, WSI (unmodified coating) exhibits the lowest ∣*Z*∣_0.1 *Hz*_ value of 6.06×10^4^ Ω⋅cm^−2^, confirming the barrier effect of WSI. Notably, CG15 achieves the highest ∣*Z*∣_0.1 *Hz*_ value, surpassing WSI by two orders of magnitude. Compared to the polyaniline/graphene oxide-modified epoxy-acrylic coatings reported by Yang et al. [26], the coatings developed in this work demonstrate superior corrosion resistance.

The results indicate that at low WSI loadings, the microscopic lamellar structure formed by graphene oxide is insufficiently dense to establish a barrier layer. As the doping concentration increases, corrosion resistance improves due to enhanced barrier properties. However, when the doping concentration exceeds 0.15%, electrical coupling between the matrix and agglomerated surface regions accelerates corrosion by providing pathways for corrosive medium penetration.

### 3.5. Corrosion Mechanism

Marine equipment operates extensively in high-salt, high-humidity seawater environments, where long-term reliability hinges on understanding coating degradation dynamics. To address this, systematic investigation of corrosion mechanisms in simulated marine conditions and the establishment of quantitative models linking accelerated aging tests to actual service lifetimes are critical for optimizing coating design and ensuring lifecycle performance. Our immersion testing protocol aligns with the core principles of ASTM D870-15 [27] (water resistance testing) and ISO 20340 [28] (cyclic marine exposure), focusing on replicating real-world environmental stressors.

**Figure 8:**
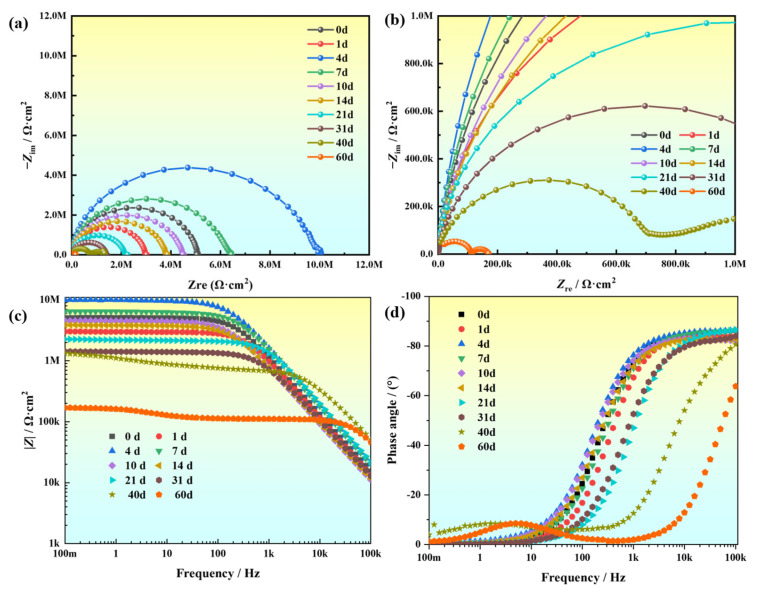
AC impedance spectra of CG15 coating immersed in artificial simulated seawater for 60 days: (**a**) Nyquist diagram; (**b**) partial magnification of Nyquist diagram; (**c**) Bode map amplitude and frequency curve; (**d**) phase–frequency curve of Bode diagram.

In order to further analyze the corrosion failure process and corrosion rule of KCG15 composite coating, a 60-day immersion experiment was carried out in artificial simulated seawater, and the AC impedance spectrum of KCG15 at different corrosion periods (0d, 1d, 4d, 7d, 10d, 14d, 21d, 31d, 40d, 60d) was measured by the electrochemical workstation. The corrosion process of the coating is described through the analysis of the AC impedance spectrum. Figure 8a–d shows the Nyquist diagram, the local magnification diagram of the Nyquist diagram, the Bode modulus diagram, and the Bode frequency angle diagram of the coating soaked in seawater for 60 days, respectively.

According to the Nyquist diagram (Figure 8a), the arc of bulk react–resistance decreases first, then increases, and then decreases with the increase in time. Three stages of the corrosion process of the coating in artificial seawater are deduced: the early corrosion stage (0~1d), shielding effect stage (1~4d), and late corrosion stage (7~60d). At the initial stage of immersion (1d), the arc radius of the bulk reactance decreases, and the low-frequency impedance mode value |Z|_0.1Hz_ decreases from the initial 4.99 × 10^6^ Ω⋅cm^2^ to 2.98 × 10^6^ Ω⋅cm^2^. The phase angle of the Bode diagram decreases, which can be attributed to the water absorption of the coating. The electrolyte now penetrates the metal/coating interface through micropores to form an ion transport channel, causing local microcell corrosion. On the fourth day of immersion, the arc radius of the capacitance react–resistance reaches its peak, and the low-frequency impedance mode value |Z|_0.1Hz_ reaches its peak value of 9.71 × 10^6^ Ω⋅cm^2^. The impedance phase angle curve presents a single time constant, the coating capacitance decreases from 2.23 × 10^−10^ to 2.07 × 10^−11^ F·cm^−2^, and the EIS phase angle shifts to the left. The increase in CPE index *N*_coat_ from 0.83 to 0.95 reflects the interfacial double layer reconfiguration, indicating the electrochemical passivation of the coating. During the late-stage failure (7–60 days), prolonged immersion led to the dissolution of corrosion products within coating pores and the precipitation of free carboxylated graphene oxide sheets. Consequently, the bulk resistance arc radius and |Z|_0.1Hz_ progressively declined. By Day 60, the Nyquist plot exhibited a double-layer capacitive arc (Figure 8a), indicative of two distinct electrochemical interfaces: (1) the corroding metal substrate and (2) the porous, degraded coating layer. This dual-arc behavior correlates with the impedance modulus, reaching its minimum value of 1.13 × 10^5^ Ω·cm^2^ (a 97.8% reduction from the initial value), and the phase angle curve broadening into dual time constants. The formation of this double-layer response confirms severe coating delamination and the establishment of parallel conductive pathways, accelerating substrate corrosion. The near-complete loss of protective capacity underscores the critical need for enhanced nanofiller dispersion or interfacial modification in future coating designs.

**Figure 9:**
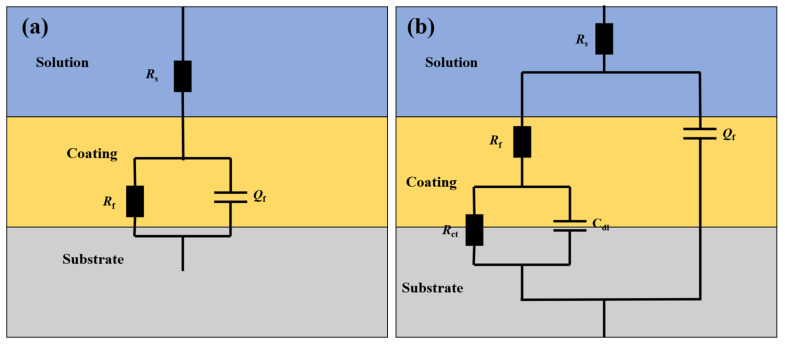
Equivalent circuit diagram of the long-term corrosion pattern of KCG15 composite coating in seawater. (**a**) Pre-corrosion stage of the coating (0~4d); (**b**) post-corrosion stage of the coating (7~60d).

According to the impedance spectral characteristics of the composite coating and the structural characteristics of the composite coating, as well as combining the results of existing studies, the equivalent circuit model of the KCG15 coating fitted in artificial simulated long-term corrosion in seawater is adopted in Figure 9 for the equivalent circuit model of KCG15 coating in the artificial simulation of long-term corrosion fitted in seawater; in Figure 9a for the pre-corrosion (0~4d) fitted circuit, whose circuit descriptor code CDC is *R*_s_(*Q*_f_ *R*_f_); and in Figure 9b for the post-corrosion (7~60d) fitted circuit, whose circuit descriptor code CDC is *R*_s_(*Q*_f_(*R*_f_(*C*_dl_*R*_ct_)))), where *R*_s_ is the solution resistance, *R*_ct_ is the charge transfer resistance of the coating to the metal substrate, *C*_dl_ represents the double capacitance associated with this charge accumulation, *R*_f_ represents the coating resistance, and *Q*_f_ denotes the coating resistance-capacitance. The values change with the penetration of the corrosive medium. The electrochemical impedance data of the coatings at each corrosion stage in artificial seawater were fitted to equivalent circuits by Zsimpwin software, and the specific parameters are listed in Table 3.

**Figure 10:**
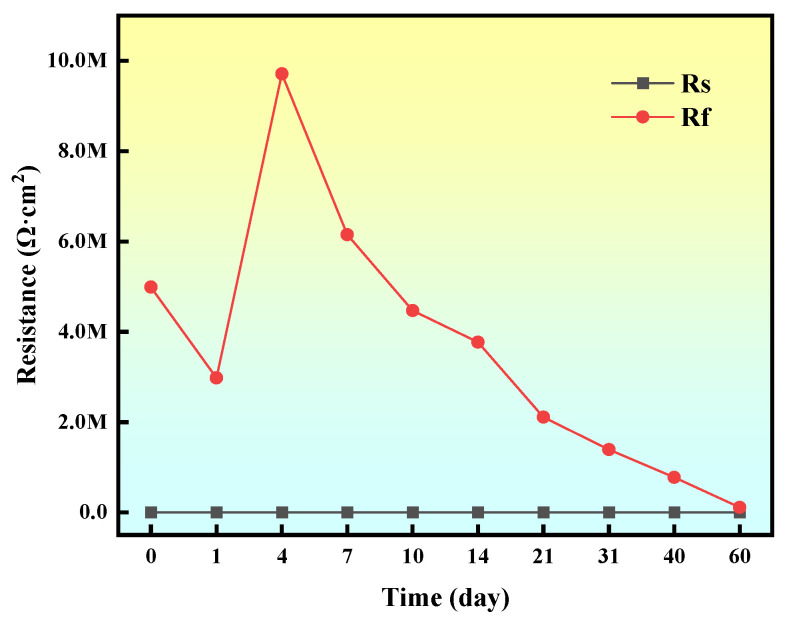
Trends of *R*_s_ and *R*_f_ in equivalent circuit diagrams.

Based on the equivalent circuit fitting results, the temporal evolution of coating resistance (*R*_f_) and solution resistance (*R*_s_) was quantitatively mapped, as illustrated in Figure 10. Analysis reveals that Rs remained relatively stable throughout the immersion period, while *R*_f_ exhibited a distinct three-stage dynamic evolution: (1) an initial sharp decline in *R*_f_, attributed to rapid electrolyte penetration and accelerated corrosion kinetics; (2) a transient rebound phase resulting from the localized barrier effect of accumulated corrosion products; and (3) a secondary reduction phase caused by progressive coating delamination and structural failure. This triphasic behavior aligns closely with electrochemical impedance spectroscopy (EIS) observations, validating the predictive capability of the proposed equivalent circuit model in describing corrosion dynamics. Crucially, the high degree of consistency between fitted and experimental data (*R*^2^ > 0.99) further corroborates the model’s reliability in simulating interfacial degradation processes under marine conditions.

### 3.6. Failure Mechanism Analysis

**Figure 11:**
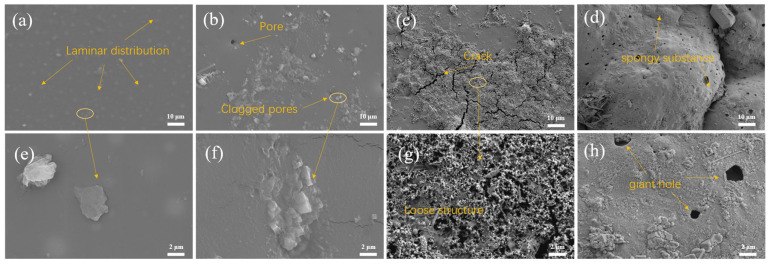
SEM surface micrographs of KCG15 coatings immersed in artificially simulated seawater environments for 0d (**a**), 4d (**b**), 31d (**c**), and 60d (**d**) and localized enlarged images of 0d (**e**), 4d (**f**), 31d (**g**), 60 (**h**).

Figure 11 presents scanning electron microscopy (SEM) images of the KCGO-15 coating after immersion in artificial seawater for varying durations (0, 4, 31, and 60 days). Panels (a), (b), (c), and (d) depict the surface morphologies at 0, 4, 31, and 60 days, respectively, while panels (e), (f), (g), and (h) show corresponding magnified views. As illustrated in Figure 11a, the pristine coating (Day 0) exhibits uniformly distributed lamellar graphene oxide (GO) sheets within the matrix, creating a tortuous diffusion pathway that impedes corrosive agent penetration to the substrate, thereby delaying corrosion initiation. On Day 4 (Figure 11b), the localized exfoliation of CGO nanosheets, corrosion product precipitation, and pore occlusion are observed. These microstructural alterations—attributed to the synergistic barrier effect of exfoliated CGO sheets and corrosion product deposits—correlate with the maximal impedance value recorded at this stage. However, prolonged immersion to Day 31 (Figure 11c) induces the formation of corrosion-induced microcracks. High-magnification imaging (Figure 11g) reveals honeycomb-like porous structures and corrosion product agglomerates adjacent to cracks, resulting from progressive coating degradation. The infiltration of seawater into these interconnected pores enhances ionic conductivity across the coating, leading to a sustained decline in coating resistance and corrosion protection efficacy. Despite severe structural compromise, the coating retains partial barrier functionality, preventing complete substrate exposure. After 60 days (Figure 11d,h), severe foam-like blistering and interconnected pore networks develop across the coating surface, resulting in the complete loss of structural integrity and substrate protection. This morphological deterioration aligns with the electrochemical data, confirming the coating’s ultimate failure to shield the substrate under prolonged immersion.

**Figure 12:**
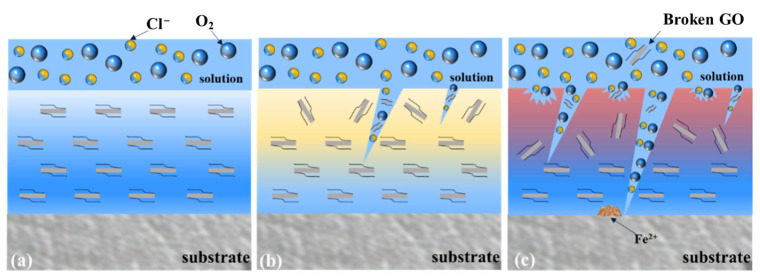
Corrosion mechanism diagram of KCG15 coating in simulated seawater. (**a**) Early stage of immersion; (**b**) middle stage of immersion; (**c**) late stage of immersion.

The integrated analysis of electrochemical impedance spectroscopy and microstructural characterization reveals the failure mechanism of the coating in simulated seawater, as schematically illustrated in Figure 12. The degradation process is distinctly categorized into three sequential phases: the Permeation Stage, the Blockage Stage, and the Failure Stage. During the initial immersion phase, the uniform dispersion of carboxyl-functionalized graphene oxide (CGO) within the coating matrix effectively obstructs the diffusion pathways of corrosive species such as Cl^−^, endowing the system with high-impedance characteristics. In the intermediate phase, water absorption saturation triggers the synergistic occlusion of interfacial pores by corrosion products and exfoliated CGO nanosheets, establishing a dynamic equilibrium that transiently preserves coating integrity. However, this phase concurrently initiates localized micro-galvanic corrosion due to electrochemical potential disparities at heterogeneous interfaces. Subsequently, prolonged immersion culminates in the dissolution of corrosion products and degradation of the silicone polymer network, manifesting as surface microcracks and a marked reduction in coating resistance. This structural compromise facilitates electrolyte permeation to the substrate, signifying the onset of protective function failure, though residual barrier properties persist to delay catastrophic corrosion.

### 3.7. Atmospheric Exposure Experiment

**Figure 13:**
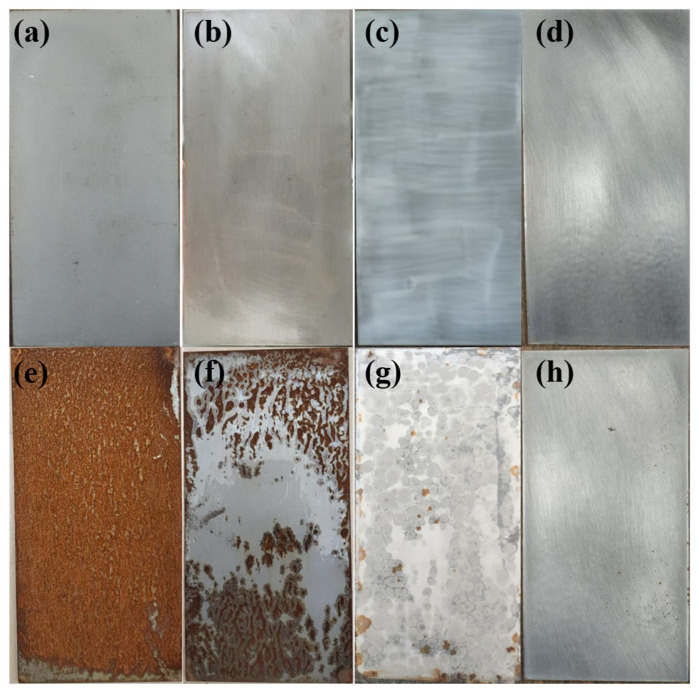
Comparison between before and after atmospheric exposure. Original macroscopic morphology of (**a**) substrate; (**b**) bisphenol A epoxy resin; (**c**) commercial waterborne paint; and (**d**) KCG15 coating. After the exposure experiment: (**e**) substrate; (**f**) epoxy resin; (**g**) commercial waterborne paint; and (**h**) KCG15.

Shown in Figure 13a–h are the before and after pictures of the Q235 steel plate, bisphenol A epoxy resin, commercial waterborne paint, and KCG15 coating placed in the atmospheric environment (longitude: 122, latitude: 31) for 180 days (13 June 2024, 12 December 2024) for the atmospheric exposure experiments to judge the use of the coating in the atmospheric environment. It can be seen that the Q235 steel plate exposed to the atmosphere shows apparent corrosion and surface roughness. The bisphenol A epoxy resin surface has an apparent dissolution cracking phenomenon, which can be attributed to the epoxy resin under ultraviolet radiation causing a photo-oxidation reaction that leads to surface degradation and chalking and rainwater infiltration of the resin inside, resulting in a decline in the performance of the material. After atmospheric exposure experiments, waterborne commercial paints showed significant discoloration, rusting, and watermarks. This is because waterborne commercial paint coatings do not adhere well to the substrate and have poor weather resistance. Rainwater penetrates the interior of commercial waterborne paints, and watermarks are formed when the weather clears up and cannot be wholly volatilized. The KCG15 coatings have the best protection for metal substrates, and only slight discoloration and a small number of grey spots on the surface of the coatings can be attributed to the KCG15 coatings causing the degradation and chalking of the surfaces. This can be attributed to the fact that the KCG15 coating has better densification and adhesion, and the water contact angle is greater than 90°, which can effectively block the penetration of water and pollutants.

## 4. Conclusions

This study successfully developed a carboxyl-functionalized graphene oxide (CGO)-modified waterborne epoxy–silicone resin composite coating, achieving a marine anti-corrosion system that integrates environmental compatibility with long-term protective performance. Key conclusions are as follows:

1. At an optimized CGO loading of 0.15 wt.%, the composite coating exhibited a corrosion current density (*I*_corr_) of 4.37 × 10⁻⁸ A/cm^2^, representing a two-orders-of-magnitude reduction compared to the pristine resin. The low-frequency impedance modulus (∣*Z*∣_0.1Hz_) reached 9.71 × 10^6^ Ω·cm^2^, demonstrating significantly improved barrier efficacy against Cl^−^ penetration. This is attributed to the tortuous pathway effect created by CGO’s lamellar structure within the resin matrix.

2. The KCG15 coating achieved a 97% inhibition rate against sulfate-reducing bacteria (SRB), with biofilm coverage reduced to <3%. The antibacterial mechanisms involve the physical disruption of bacterial cell membranes by CGO’s sharp edges and electrostatic repulsion from negatively charged carboxyl groups (-COOH) on CGO surfaces.

3. A 60-day seawater immersion test revealed three distinct corrosion phases: Permeation Phase (0–1 day): Rapid electrolyte infiltration caused impedance decline. Blockage Phase (1–4 days): Synergistic pore occlusion by corrosion products and exfoliated CGO temporarily restored impedance. Failure Phase (7–60 days): Progressive structural degradation transitioned from microcrack networks (Day 30) to foam-like porous blistering (Day 60). This morphological collapse formed interconnected ionic channels, reducing |Z|_0.1Hz_ to 1.13 × 10^5^ Ω·cm^2^. Despite the complete loss of structural integrity, residual impedance remained 1.4-fold higher than the pristine resin (WSI: 8.2 × 10^4^ Ω·cm^2^), demonstrating delayed but irreversible barrier failure.

4. After 180 days of atmospheric exposure, the KCG15 coating exhibited only minor surface discoloration with no rust spots or delamination, outperforming commercial epoxy and waterborne coatings. Its hydrophobicity (>90° contact angle) and interfacial adhesion strength (3.67 MPa) ensured stability in harsh marine environments, meeting long-term protection requirements for deep-sea infrastructure.

## Figures and Tables

**Table 1:** Chemical composition of artificial simulation seawater (g/L).

NaCl	MgCl_2_	Na_2_SO_4_	CaCl_2_	KCl	SrCl_2_	NaHCO_3_	KBr	H_3_BO_3_	NaF
24.530	5.200	1.090	1.160	0.695	0.025	0.201	0.101	0.027	0.003

**Table 2:** Polarization curve fitting parameters of coatings.

Sample	*E*_corr_/mV	*I*_corr_/A⋅cm^−2^	*V*_corr_/mm⋅a^−1^
WSI	−291	2.05 × 10^−6^	4.77 × 10^−1^
KCG5	−173	9.86 × 10^−7^	1.26 × 10^−2^
KCG10	−140	7.17 × 10^−8^	1.39 × 10^−3^
KCG15	−101	4.37 × 10^−8^	3.41 × 10^−4^
KCG20	−165	9.06 × 10^−8^	3.47 × 10^−3^
KCG25	−226	2.44 × 10^−7^	6.41 × 10^−3^

**Table 3:** EIS fitting results for coating equivalent circuits.

Time	*R*_s_ (Ω⋅cm^2^)	CPE_-t_ (Ω^−1^cm^−2^s^n^)	*N*_coat_	*R*_f_ (Ω⋅cm^2^)	C_dl_ (F⋅cm^−2^)	*R*_ct_ (Ω⋅cm^2^)	Goodness of Fit
0d	732.5	2.08 × 10^−10^	0.95	4.99 × 10^6^			8.96 × 10^−5^
1d	530.8	2.23 × 10^−10^	0.83	2.98 × 10^6^			8.63 × 10^−5^
4d	610.4	2.07 × 10^−11^	0.95	9.71 × 10^6^			1.35 × 10^−4^
7d	538.9	2.33 × 10^−10^	0.73	6.15 × 10^6^	7.81 × 10^−11^	2.12 × 10^5^	1.32 × 10^−4^
10d	333.7	3.59 × 10^−10^	0.93	4.47 × 10^6^	1.83 × 10^−10^	5.15 × 10^4^	6.53 × 10^−5^
14d	185.1	3.58 × 10^−10^	0.93	3.77 × 10^6^	3.56 × 10^−7^	5.71 × 10^4^	3.82 × 10^−5^
21d	308.4	1.24 × 10^−10^	0.96	2.11 × 10^6^	1.25 × 10^−7^	9.54 × 10^4^	1.15 × 10^−4^
31d	100	3.29 × 10^−9^	0.93	1.39 × 10^6^	2.74 × 10^−8^	1.85 × 10^4^	4.12 × 10^−5^
40d	285	1.42 × 10^−11^	0.89	7.77 × 10^5^	1.26 × 10^−6^	4.31 × 10^4^	2.53 × 10^−4^
60d	209	5.17 × 10^−11^	0.8	1.13 × 10^5^	6.36 × 10^−7^	5.15 × 10^4^	1.53 × 10^−5^

## Data Availability

Data will be made available on request.
